# Survival, Mortality Predictors, and Morbidity in Extremely Low Birth Weight Neonates: A Retrospective Cohort Study at a Tertiary Hospital in the Eastern Cape, South Africa

**DOI:** 10.3390/children13030317

**Published:** 2026-02-25

**Authors:** Sithembinkosi Manyoni Gonya, Kim Harper, Isabel Michaelis

**Affiliations:** Department of Paediatrics, Faculty of Medicine and Health Sciences, Walter Sisulu University, Private Bag X1, Mthatha 5117, South Africa; kharper@wsu.ac.za

**Keywords:** extremely low birth weight (ELBW), neonatal mortality, survival outcome, resource-limited settings, extreme prematurity, South Africa

## Abstract

**Background**: Extremely low birth weight (ELBW) neonates (<1000 g) contribute significantly to global neonatal and under-five mortality, with heightened vulnerability in resource-limited settings. **Objectives**: The overall aim of this manuscript is to evaluate the survival outcomes and associated factors among ELBW infants in a resource-limited tertiary care setting in South Africa. **Methods**: This retrospective cohort study examined survival outcomes, causes of death, and associated morbidities among 192 ELBW infants admitted to Frere Hospital, South Africa (SA), between January 2020 and February 2025. Kaplan–Meier survival analysis and multivariable Poisson regression were used to identify predictors of mortality. **Results**: Overall, 42% of neonates survived to discharge. Common morbidities included respiratory distress syndrome (78%) and extreme prematurity (30%). Extreme prematurity (<28 weeks) was associated with a markedly increased risk of mortality (adjusted IRR = 0.20; 95% CI: 0.08–0.53; *p* < 0.001), while caesarean section conferred a protective effect compared to vaginal delivery (adjusted IRR = 0.38; 95% CI: 0.23–0.64; *p* < 0.001). **Conclusions**: The findings highlight that strengthened antenatal care, consistent neonatal resuscitation, and equitable intensive care remain essential. Policy-driven investment in surfactant therapy, CPAP, and infection control is critical; without such measures, ELBW infants’ mortality will continue to be disproportionately high in resource-limited settings.

## 1. Introduction

Extremely low birth weight (ELBW), defined as a birth weight of less than 1000 g, remains a significant contributor to neonatal mortality globally and has a considerable impact on under-five mortality rate (U5MR) [1,2]. As a global health indicator, U5MR reflects child survival and broader socioeconomic conditions. Despite notable improvements in ELBW survival in high-income countries (HICs), ELBW-related deaths continue to disproportionately affect low- and middle-income countries (LMICs) [1]. However, there are few studies on the mortality of ELBW in Africa and South Africa (SA) [1,3,4]. Birth weight classifications—low birth weight (<2500 g), very low birth weight (<1500 g), and ELBW (<1000 g)—are internationally recognised standards [5]. Weight categorisation remains a cornerstone in the management of ELBW infants, as survival and morbidity outcomes differ sharply across defined weight bands. Recent global guidance from the World Health Organization (WHO) emphasises stratification by birth weight and gestational age (GA) to guide intervention thresholds and parental counselling [5,6]. Infants below 750 g continue to demonstrate the highest mortality and require aggressive interventions such as early surfactant and neonatal intensive care unit (NICU) care, while those 750–999 g show improved survival but remain at high risk of respiratory distress [7]. Infants >1000 g generally have better outcomes [8].

ELBW infants may be born preterm (<37 weeks of gestation) or at term (>37 weeks), with preterm births further categorised as extremely (<28 weeks), very (28–<32 weeks), and moderate/late (32–<37 weeks) [5,9,10]. GA categorization is fundamental in the management of ELBW infants as it provides a more accurate predictor of survival and mortality, synergistically with birth weight. Infants born at ≤25 weeks’ gestation demonstrate the highest mortality and are at greatest risk of complications such as respiratory distress syndrome (RDS), intraventricular haemorrhage (IVH), and impaired thermoregulation, necessitating aggressive NICU interventions [5]. Those born at 26–28 weeks show improved survival but remain vulnerable, requiring immense care, selective surfactant administration, and early continuous positive pressure (CPAP) initiation [11]. By 29–32 weeks, organ maturity improves significantly, resulting in better outcomes due to greater organ maturity (lung, brain, and gastrointestinal systems) [5,12]. Recent global guidelines emphasise that gestational age stratification is essential not only for tailoring respiratory and nutritional strategies but also for distinguishing preterm infants from small-for-gestational-age neonates, thereby guiding parental counselling and resource allocation [13]. Collectively, GA and weight categorisation provide a universal framework for risk assessment and intervention planning, ensuring that ELBW infants receive care aligned with their developmental maturity and weight.

In 2020, an estimated 13.4 million preterm births occurred globally, with national rates ranging from 4 to 6% [5]. Prematurity is the predominant factor in ELBW admissions, with the highest mortality observed in infants born before 28 weeks of gestation [1].

Sub-Saharan Africa and South Asia account for most preterm births, where complications related to prematurity are a leading cause of neonatal death [14]. Survival disparities between HICs and LMICs are stark: while 9 in 10 ELBW infants survive in HICs, only 1 in 10 do so in LMICs. In response, WHO has issued updated guidelines to improve ELBW outcomes in resource-limited settings, including maternal and neonatal care recommendations. Survival is defined as the duration from birth to discharge, while the mortality rate refers to the number of deaths within a defined period [15,16]. Birth weight and gestational age (GA) are consistently identified as independent predictors of neonatal mortality, with ELBW infants facing the highest risk regardless of term status [17]. In HICs, survival rates for ELBW infants have improved markedly due to evidence-based interventions such as antenatal corticosteroids (ACS), surfactant replacement therapy (SRT), and early use of CPAP [18]. These advances have led to survival rates exceeding 85% in some neonatal intensive care units (NICUs) [18,19]. In contrast, LMICs continue to report ELBW survival rates below 50%, mainly due to limited access to these interventions [1,18,20]. Similarly, a global meta-analysis of 22,278 ELBW infants reported an overall survival rate of 34%, with rates as low as 18% in LICs and 39% in upper-MICs [21]. In SA, survival rates vary widely across provinces and institutions. At Tygerberg Hospital in the Western Cape, a 2021 study reported a survival-to-discharge rate of 63.3%, attributed to the selective use of SRT and nasal CPAP [22]. In contrast, a study from Bloemfontein found that only 23.3% of ELBW neonates were discharged alive [1]. A single-centre study in the Eastern Cape reported a 53.5% ELBW survival rate, notably lower than urban centres like Tygerberg [23].

In high-income countries, advanced care has lowered survival thresholds to around 24–25 weeks, while in South Africa and other low-resource settings it remains above 27 weeks [24]. In South Africa, management of preterm birth follows national maternal and perinatal care guidelines, emphasising timely interventions to improve neonatal survival while safeguarding maternal health. These include early diagnosis of preterm labour, corticosteroid administration to the mother (24–34 weeks), short-term tocolysis to allow steroid effect, and magnesium sulphate for neuroprotection before 32 weeks. The latter is usually not available in all delivery centres. Infection control through antibiotics in cases of premature rupture of membranes (PPROM) or suspected intrauterine infection, alongside referral to facilities with neonatal intensive care capacity, are recommended. Delivery planning is individualised and guided by gestational age of the foetus and thus its viability (>27 weeks locally), taking maternal and foetal factors into consideration, and thus trying to combine global best practice and adaptation to resource constraints [24,25].

In South Africa, preterm caesarean section is most often indicated for severe maternal complications such as preeclampsia/eclampsia and antepartum haemorrhage. Foetal indications include intrauterine growth restriction (IUGR), foetal distress, and malpresentation. Other contributing factors include multiple-pregnancy complications, preterm prelabour rupture of membranes (PPROM) with infection or foetal compromise, and failed induction of labour. These indications highlight the balance between maternal safety and neonatal survival, with decisions shaped by gestational age viability (>27 weeks) and neonatal intensive care availability [26].

South African studies highlight persistent barriers, including inadequate ACS coverage, delayed respiratory support, and restricted NICU admission for infants [18,20,27]. In SA, NICU admission is rationed due to limited beds, equipment, and staff. Public hospitals often set a cut-off of 1000 g birth weight for NICU admission, meaning most ELBW infants are excluded. This contributes to poorer survival outcomes compared to high-income countries [22].

Leading causes of death among ELBW infants include extreme prematurity, multi-organ immaturity, respiratory distress syndrome (RDS), and neonatal sepsis [1,14]. Access to advanced ventilation support—such as conventional ventilation, high-frequency oscillatory ventilation (HFOV), and CPAP—is critical for managing RDS but remains limited in many South African facilities [20,28]. These constraints underscore the vulnerability of ELBW infants in resource-limited settings, where gaps in neonatal care compound structural immaturity and infectious complications [20]. This study evaluated survival outcomes among ELBW infants admitted to the neonatal unit at Frere Hospital, East London, SA. Specifically, it sought to determine the proportion of ELBW infants who survived until discharge, identify primary causes of death, and explore neonatal factors—such as gestational age, birth weight, associated morbidities, and mode of delivery—that may correlate with survival or mortality. By documenting these outcomes and contributory factors, the study provides insight into the challenges and opportunities for improving ELBW care in a tertiary-level neonatal setting.

Furthermore, beyond immediate neonatal complications, long-term outcomes such as risk of readmission are increasingly recognised as critical to the survival and quality of life of extremely low birth weight (ELBW) infants. Recent work by [29] highlights the burden of neonatal readmissions and underscores the importance of structured follow-up programmes for ELBW survivors. Incorporating these insights strengthens the argument for continuity of care and comprehensive monitoring strategies that extend beyond the neonatal period.

## 2. Materials and Method

### 2.1. Study Design and Setting

This retrospective study was conducted at Frere Tertiary Public Hospital in East London, Eastern Cape, South Africa. The neonatal high-care unit at Frere Hospital offers both level II and III care services, serving as a referral centre for the surrounding district hospitals. The study period was from January 2020 to February 2025.

### 2.2. Study Population

All infants admitted to the neonatal unit during the study period with a birth weight of less than 1000 g were eligible for inclusion. Infants were excluded if they had incomplete medical records or if folders were inaccessible due to a poor filing system.

### 2.3. Data Collection

Using a standardised data collection sheet, data were extracted from neonatal admission registers, patient files, and discharge summaries. Variables collected included birth weight, gestational age, sex, mode of delivery, APGAR scores, antenatal corticosteroid administration, surfactant therapy, respiratory support modalities, documented morbidities (e.g., respiratory distress syndrome, sepsis), and survival status at discharge. Neonatal records with incomplete data for critical variables, including birth weight, gestational age, and survival status, were excluded from the analysis. Variables with minimal missingness (<5%) were retained without imputation. The data were collected and stored on the researcher’s computer, password-protected. Additionally, the data was encrypted for further security.

### 2.4. Definitions

Birth weight categories were based on WHO standards: extremely low birth weight (<1000 g). Gestational age was determined by clinical assessment. Survival to discharge was defined as the infant being alive at the time of hospital discharge. Mortality was defined as death occurring during the neonatal admission.

### 2.5. Outcome Measures

The primary outcome was survival to discharge among ELBW infants. Secondary outcomes included causes of death, frequency of associated morbidities, and correlations between survival and neonatal factors such as gestational age, birth weight, and mode of delivery.

### 2.6. Data Analysis

Descriptive statistics were utilised to summarise the dataset using the Microsoft 2024 (USA) application. Descriptive statistics were presented in tables, bar graphs, and charts. Continuous variables were summarised using means with standard deviations (SDs) for parametric data, or medians with interquartile ranges (IQRs) for non-parametric data. Categorical variables were presented as frequencies and percentages, and comparisons were conducted using the chi-squared test, depending on data suitability. All statistical tests were two-tailed, and a *p*-value less than 0.05 was considered to indicate statistical significance. Survival analysis was conducted using Kaplan–Meier curves to assess the survival rate and associated factors. Poisson regression was adopted to adjust for multivariable variables contributing to mortality and the causes of death in ELBW infants.

### 2.7. Ethical Considerations

Ethical approval was received from the Walter Sisulu University (WSU) Sciences Ethics Committee board, WSU HREC 046/2025, and the Provincial Ethics Board, EC_202505_008. Permission for the study was obtained from the Chief Executive Officer of the hospital and the Head of the Department of Paediatrics. The researcher did not need informed consent since it was a retrospective study, and all participants were anonymised.

## 3. Results

### 3.1. Demographics of Infants

**Figure 1:**
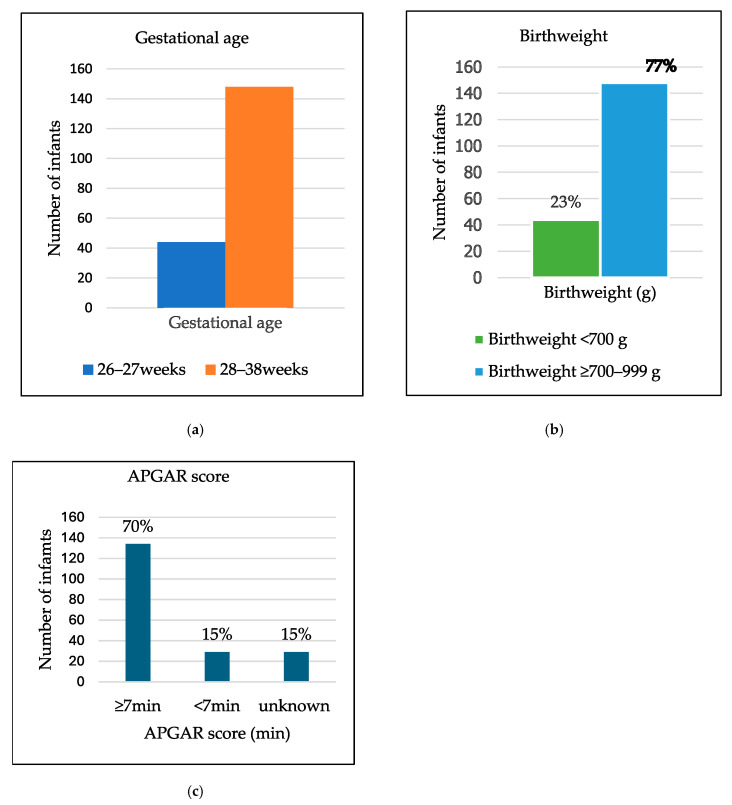
Bar graph: (**a**) Demographics of infants according to gestational age. (**b**) Pie charts of infants’ demographics by birth weight. (**c**) APGAR scores.

A total of 296 infants met the inclusion criteria; of these, 192 folders of infants < 1000 g were included in the study. Ninety-nine infants (52%) were female. One hundred and forty-eight (77%) infants had a GA above 27 completed weeks. The most significant proportion, 148 (77%) infants, weighed ≥700 g but <1000 g. A total of 134 infants (70%) had a 5 min APGARscore ≥ 7, and participants with either a 5 min APGARscore < 7 or an unknown APGARscore comprised 29 (15%) each, as indicated below in Figure 1.

**Figure 2:**
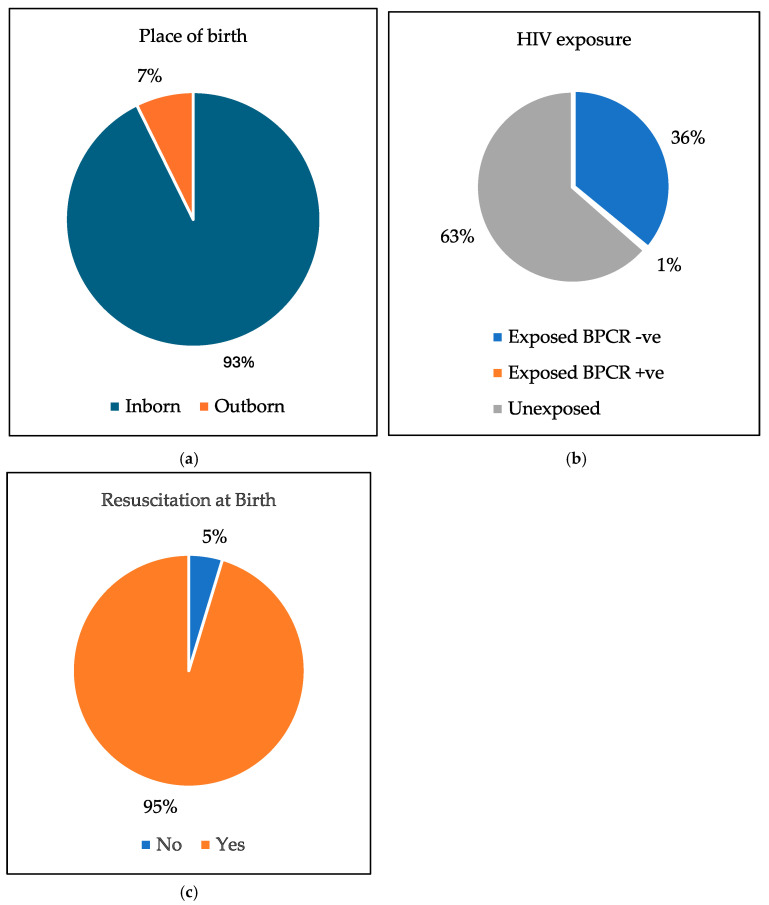
The pie charts of: (**a**) The place of birth. (**b**) HIV exposure. (**c**) Resuscitation at birth.

In this study, 178 infants (93%) were born at Frere Hospital. In terms of HIV exposure, 69 infants (36%) were exposed with a negative HIV PCR at birth, and 1 infant (0.5%) was exposed and tested positive. A significant majority of ELBW infants in the study, 183 infants (95%), required resuscitation at birth. See Figure 2.

### 3.2. Respiratory Support

**Figure 3:**
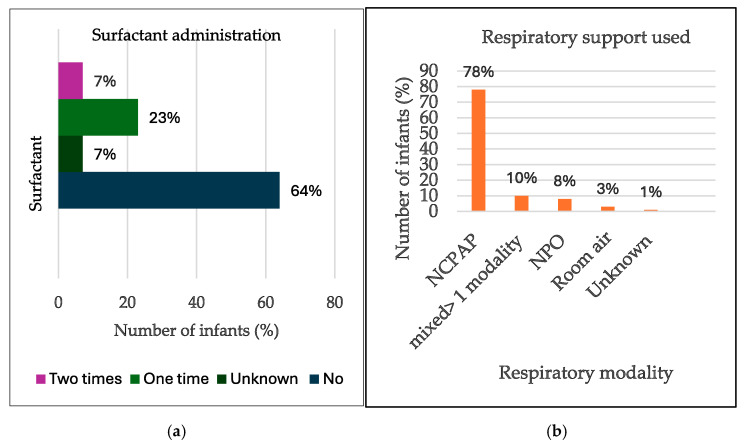
Bar graphs of: (**a**) Respiratory support. (**b**) Surfactant administered.

Regarding respiratory support, most of the infants, 185 (96.3%), received some kind of respiratory support, as shown in Figure 3 below. NCPAP alone was the intervention in 150 cases (78%), while 19 infants (10%) received >2 modalities of support. Nasal prong oxygen (NPO)-only support was provided to sixteen infants (8%), and five infants (3%) were managed on room air.

Two cases (1.0%) had unknown respiratory support modalities. A total of 44 (23%) infants received SRT once, and 13 (7%) received a second dose, while 122 infants (64%) did not receive SRT, and administration details were missing for 13 cases (7%). See Figure 3 below.

### 3.3. Survival Outcome

Of the total cohort of 192 infants, 80 infants (42%) survived their admission to the neonatal ward.

### 3.4. Morbidity Patterns

**Figure 4:**
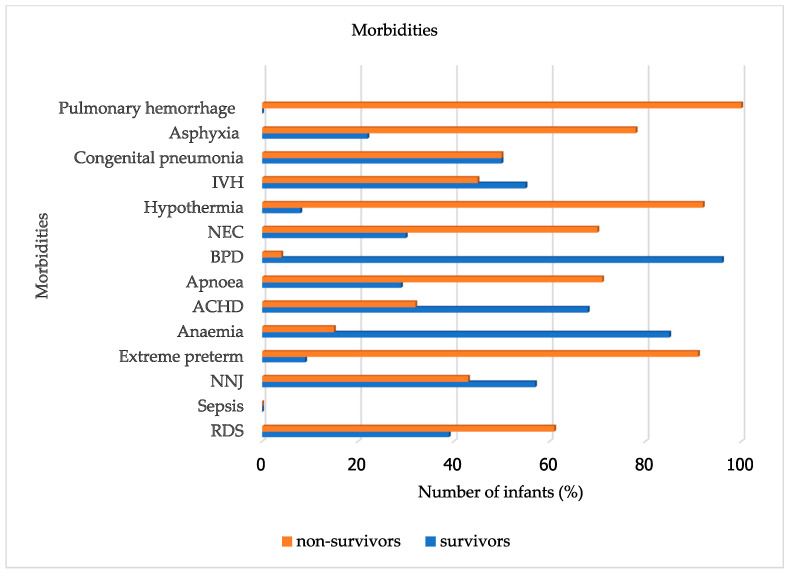
Morbidities of survivors and non-survivors.

The most frequently recorded comorbidity was RDS, affecting 150 infants (78.1%), with 61% non-survivors and 39% survivors. Sepsis was reported in 112 infants (58.3%), with almost equal survivors and non-survivors, 45% and 55%, respectively. Neonatal jaundice (NNJ) similarly had equal distribution, 43% non-survivors, and 57% survivors. ACHD acyanotic congenital heart diseases, which include patent ductus arteriosus (PDA), which is usually most common on clinical diagnosis in our settings, had 68% survivors and 32% non-survivors. Extreme prematurity was associated with the highest non-survival rate, at 91%, as shown in Figure 4.

### 3.5. Regression Analysis

**Figure 5:**
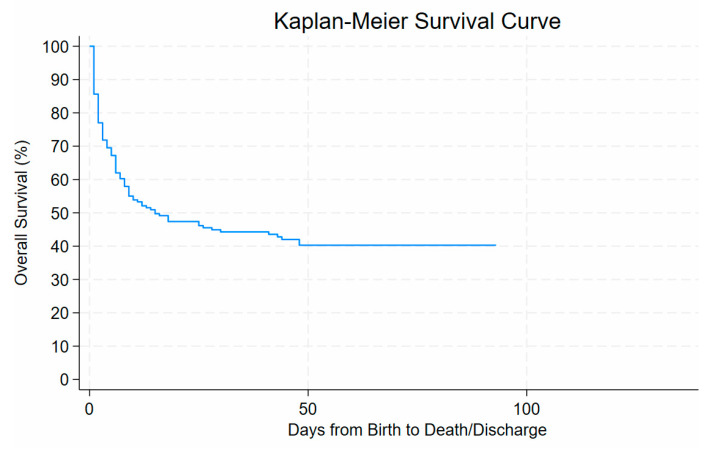
Risk of dying of ELBW related to days of life.

The Kaplan–Meier survival analysis demonstrated a steep decline in survival probability within the first few days after birth, with overall survival decreasing from 100% at day 0 to approximately 70% by day 10 of life. Survival declined gradually thereafter, reaching around 50% by day 30 of life and stabilising at approximately 40% beyond day 50. No further deaths occurred after about 90 days from birth until discharge. See Figure 5.

### 3.6. Analysis of Survivors and Non-Survivors According to Birth Weight and GA

**Figure 6:**
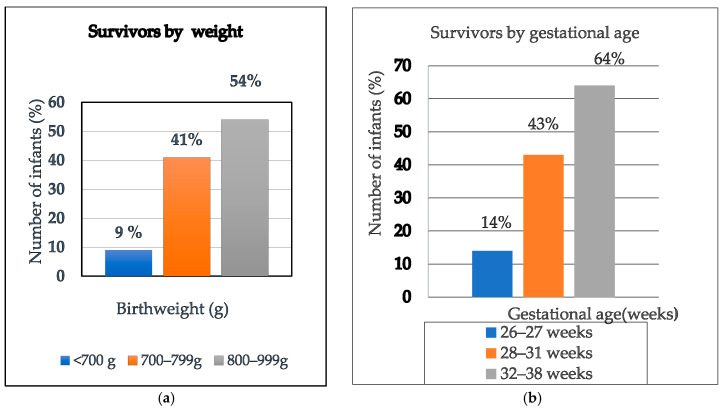
Bar graphs of (**a**) survivors by weight, (**b**) survivors by gestational age.

Birth weight and GA were strongly associated with survival outcomes (*p* ≤ 0.001). Higher weight and older GA were associated with better survival outcomes, as shown in Figure 6.

With multivariable regression, only extreme prematurity and mode of delivery showed statistically significant associations with the survival outcome (*p* < 0.001), with confidence intervals that did not cross 1. All other variables analysed had *p*-values > 0.05, indicating no statistically significant effect, as shown in Table 1.

## 4. Discussions

### 4.1. Survival and Mortality Outcomes of ELBW

This study underscores the substantial burden of mortality and morbidity among ELBW infants in a resource-limited neonatal setting. In this study, the survival rate among ELBW infants was 42%, slightly lower than the 46% reported in a comparable cohort at Frere Hospital and the 63.3% survival in a cohort of ELBW infants at Tygerberg Hospital in the Western Cape in SA [22,23]. Nevertheless, the survival rate of ELBW infants in this study was higher than that recorded in a recent prospective study in Bloemfontein of 23.3% [1]. In contrast, the survival of 42% in this study was far below that of HICs, which exceeds 85% [18,19]. Another finding, which corresponds with former studies, revealed a steep decline in survival within the first week of life, emphasising a critical window where timely interventions may yield the most significant impact on neonatal outcomes [1,21,22].

Several contextual factors likely contributed to this outcome, including the high proportion of ELBW infants, limited access to advanced neonatal intensive care interventions, and constraints in surfactant availability. In our setting, surfactant administration was guided by institutional protocols and restricted to infants >700 g and >27 weeks’ gestation, with administration further dependent on stock availability at the time of admission. These limitations reduced consistent access to therapy and may have contributed to the observed mortality.

Among infants who survived, several factors appeared to support improved outcomes: relatively higher birth weights within the ELBW range, gestational age closer to 28 weeks, initiation of respiratory support, and access to surfactant therapy when available. Survival was also more likely in infants without major comorbidities such as severe sepsis or intraventricular haemorrhage. While these associations are observational and not stratified in the dataset, they provide important insights into potential protective factors

### 4.2. Survival by Birth Weight

The findings of this study demonstrated a survival rate of only 9% in infants with birth weight < 700 g, underscoring the inferior outcomes associated with this weight category in our institution. Notably, survival improved fourfold among infants weighing ≥700 g and <800 g, indicating a significant threshold effect. However, among those with birth weights ranging from 800 g to 999 g, the increase in survival was more modest, rising by only 13% suggesting a plateau in survival gains within this higher weight bracket. The higher birth weight and higher GA in this study were strongly associated with improved survival outcomes (*p* < 0.001), aligning with findings from many other studies [22,30,31]. In our study, the smallest infant to survive until discharge weighed just 539 g and was born at 28 weeks’ gestation, illustrating the potential for survival even at the margins of viability. Recent studies have reported similar survival thresholds, with documented cases of infants weighing less than 500 g achieving survival-to-discharge outcomes [32,33]. Despite recent advances, survival remains disproportionately affected by factors such as gestational age below 28 weeks, birth weight under 800 g, lack of SRT, and delayed or inadequate respiratory support.

### 4.3. Impact of Delivery Mode

In this study, expected vaginal delivery (NVD) was associated with a significantly lower likelihood of survival when compared to C/S, with an adjusted incidence rate ratio (IRR) of 0.38 (95% CI: 0.23–0.64; *p* < 0.001). In this study, caesarean section was associated with a protective effect compared to vaginal delivery. The finding raises important questions about potential mechanisms, particularly whether differences in respiratory management strategies and/or delivery context contribute to improved outcomes. Understanding how delivery mode influences neonatal outcomes is critical for optimising perinatal care, especially in extremely low birth weight (ELBW) infants who remain at high risk of morbidity and mortality. Unfortunately, this could not be explored in this study due to the retrospective nature. Recent literature has reported similar improved neonatal outcomes with caesarean delivery in high-risk cohorts [32,33,34]. The C/S mode offers a controlled environment that minimises intrapartum hypoxia and mechanical stress [34]. The operating theatre setting ensures immediate access to advanced neonatal resuscitation, which may contribute to better APGARscores and reduced need for invasive respiratory support [34]. It remains important to mention that even though C/S is a valuable tool to improve survival of high-risk infants, it only remains so if prematurity and GA are considered and should only be considered when the mother’s or foetus’s life is at risk. The above research findings reinforce the critical role of birth weight, GA, antenatal optimisation, and specialised neonatal care in improving infant survival prospects at the limits of viability.

### 4.4. Role of APGARScore

The study’s findings showed that low APGAR scores (*p* ≤ 0.05) were also significantly associated with mortality, which was illustrated by 79% of infants with a 5 min APGAR score below 7 who died before discharge. Recent studies confirmed the high risk of death with a 5 min APGAR score of less than 5 [35,36,37]. The 5 min APGARscoring is a prognostic value and will further help with counselling the mothers about the prognosis of their infant [38,39].

### 4.5. Predominant Causes of Death

Extreme prematurity emerged as the leading cause of mortality in this cohort, with a statistically significant association (*p* ≤ 0.001) and a mortality rate of 91.4%. This was further substantiated by adjusted Poisson regression analysis, which yielded a risk ratio of 0.20 (95% CI: 0.08–0.53), indicating an 80% likelihood of death among ELBW infants, a finding congruent with reports of other South African studies [1,22]. Hypothermia also demonstrated a significant association with increased mortality, with a risk elevation of 92%, as observed by Smith and Betelew [40,41,42].

### 4.6. RDS and Mortality

Respiratory distress syndrome (RDS) was the most prevalent morbidity, affecting 78% of ELBW infants, consistent with the well-established pulmonary immaturity in this population [43]. Despite being associated with a 61% mortality rate in this study, RDS did not reach statistical significance (*p* > 0.05) as an independent predictor of mortality. This may be attributed to its strong correlation with extreme prematurity and low birth weight—factors that are themselves dominant predictors of neonatal death. In multivariate analysis, adjusting for these variables likely diminished the standalone influence of RDS. Our dataset did not capture detailed information on the timing of respiratory interventions or comparative surfactant administration between survivors and non-survivors. In our setting, surfactant administration was guided by institutional protocols and constrained by availability. Infants with RDS who weighed more than 700 g and were beyond 27 weeks’ gestation were considered eligible for surfactant therapy. However, administration was also dependent on stock availability at the time of admission, which limited consistent access. Infants <1000 g are rarely admitted to the NICU or offered invasive ventilation. Substantiating evidence from published South African and LMIC studies demonstrate the impact of delayed CPAP initiation and restricted surfactant access on ELBW survival [22]

### 4.7. Sepsis and Mortality

Sepsis was also a common finding, reported in 58% of the infants. This number affirms findings that neonatal sepsis remains a leading cause of death due to immature immune responses and prolonged hospitalisation [4]. Although sepsis is globally recognised as a significant contributor to neonatal death, in this study, it was associated with a 55% mortality rate but did not achieve statistical significance (*p* > 0.05). This contrasts with prior studies, where sepsis showed a clear independent association with neonatal mortality [1,44]. The lack of significance observed here may reflect the interplay of early diagnosis and aggressive management strategies, coupled with other severe comorbidities, particularly extreme prematurity, which may have diminished the independent predictive power of sepsis in this cohort.

### 4.8. PDA and Mortality

Patent ductus arteriosus (PDA) remains an important consideration in the care of extremely premature infants, given its potential impact on morbidity and mortality. In the present study, routine cardiac ultrasound screening for PDA was not performed, which represents a key limitation. As a result, the prevalence of PDA and its contribution to adverse outcomes could not be systematically assessed. Acknowledging this limitation is essential for transparency and helps define the scope of the findings.

## 5. Conclusions

In summary, the study highlights the persistently high mortality rate among ELBW infants in our institution, with a mortality of 91% in those weighing less than 700 g, stressing the urgent need for targeted interventions and enhancing antenatal care (ANC).

### 5.1. Recommendations

Improving ANC, optimising the timing and mode of delivery for at-risk mothers, and ensuring prompt neonatal resuscitation are critical components. Enlarging the capacity of the NICU and high-care ward can help ensure that ELBW infants receive the recommended care. Implementing structured perinatal protocols, alongside efforts to address gaps in neonatal resources and antenatal interventions, can significantly alter survival trajectories and reduce mortality rates in ELBW populations within our setting.

### 5.2. Future Studies

Future prospective and multicentre studies with broader sociodemographic data and long-term neurodevelopmental follow-up are warranted to provide a more comprehensive understanding of survival outcomes in this vulnerable population in our setting. Future studies should also integrate stillbirth to better contextualise survival outcomes at comparable gestational ages. Also, further studies are needed to explore whether differences in respiratory management and delivery context confirm the association found in our study. The importance of long-term follow-up and provision of neurodevelopmental assessments and therapies cannot be underestimated. Such approaches will provide a more complete understanding of both early and late outcomes, ultimately guiding interventions to improve survival and quality of life in this vulnerable population.

## Figures and Tables

**Table 1:** Analysis of variables from a Poisson regression analysis.

Variable	Unadjusted Model	Adjusted Model
	IRR (95% CI)	IRR (95% CI)
Extreme preterm		
No	1	1
Yes	0.15 (0.062–0.38) *	0.20 (0.08–0.53) *
Mode of delivery		
Caesarean section	1	1
NVD	0.39 (0.24–0.64) *	0.38 (0.23–0.64) *

* *p* < 0.001, IRR: incidence rate ratio, CI: confidence interval, NVD: normal vaginal delivery, *p*-values from Poisson regression analysis.

## Data Availability

The data supporting the findings of this study on ELBW infants are available from the corresponding authors upon reasonable request. Due to institutional policies and ethical considerations regarding patient confidentiality, the dataset is not publicly accessible.

## Abbreviations

ACS	Antenatal corticosteroids
ANC	Antenatal care
BPD	Bronchopulmonary dysplasia
C/S	Caesarean section
EC	Eastern Cape
ELBW	Extremely low birth weight
GA	Gestational age
HICs	High-income countries
HIV	Human Immunodeficiency Virus
IVH	Intraventricular haemorrhage
LICs	Low-income countries
MICs	Middle-income countries
NEC	Necrotising Enterocolitis
NICU	Neonatal intensive care unit
RDS	Respiratory distress syndrome
SA	South Africa
SRT	Surfactant replacement therapy
VLBW	Very low birth weight
WHO	World Health Organization
